# On the RET Rearrangements in Chernobyl-Related Thyroid Cancer

**DOI:** 10.1155/2012/373879

**Published:** 2011-11-20

**Authors:** Sergei V. Jargin

**Affiliations:** Department of Pathology, Peoples' Friendship University of Russia, Clementovski Per 6-82, Moscow 115184, Russia

## Abstract

There is a consensus that Chernobyl accident has induced thyroid cancer increase in children and adolescents. The UNSCEAR report concluded that no somatic disorders other than thyroid cancer were caused by radiation exposure due to the accident except for acute radiation sickness occurred to the people within the Power Plant at the time of the accident. A hypothesis is discussed in this paper that the increase of thyroid cancer was caused predominantly by the screening, overdiagnosis, and registration of nonirradiated persons as Chernobyl victims. A mechanism of thyroid cancer overdiagnosis is described that can be active even today, causing hypertherapy. Older neglected tumors found by the screening shortly after the Chernobyl accident or brought from noncontaminated areas were misclassified as aggressive radiation-induced cancers. Therefore, supposed markers of the radiation-induced thyroid cancer, such as the RET rearrangements, are probably associated with disease duration and tumor progression. The screening effect is obviously dependent on the basis level of medical surveillance: the higher the level, the smaller the screening effect. Absence of any significant increase of thyroid cancer after the Fukushima accident in spite of the vigorous screening would certify the high level of health care in Japan especially for children.

In some publications [1, 2], cause-effect relationships between radiation, certain genetic abnormalities, and incidence increase of the post-Chernobyl thyroid cancer (TC) are treated as proven facts. It was even assumed that elevated radiation background and medical exposures could have contributed to the TC incidence increase in some countries [3]. The possibility that the post-Chernobyl TC increase was largely caused by factors irrelevant to ionizing radiation was discussed previously [4–9]. Outdated equipment of laboratories in the early 1990s and insufficient quality of histological specimens hindered reliable histopathological examination. Access to foreign professional literature has been limited in the former Soviet Union. High tumor expectancy after the accident contributed to overdiagnosis of cancer. Appearance of advanced tumors shortly after the accident can be explained by a screening effect with detection of old undiagnosed cancers, and by the fact that patients from noncontaminated areas were registered as Chernobyl victims. Some of such cases were classified as aggressive radiogenic cancers developing after a short latency. Accordingly, some features of supposedly radiogenic TC must characterize, on average, a later stage of the tumor progression. For example, chromosomal rearrangements of the tyrosine kinase proto-oncogenes *RET*, the *RET/PTC3* in particular, found in high proportion in papillary TC (PTC) of patients exposed during childhood and adolescence [10, 11], were discussed as possible markers of radiogenic cancer [2, 12]. Over time, percentage of tumors with *RET* rearrangements declined, while among *RET*-positive tumors the percentage with *RET/PTC1* increased and *RET/PTC3* decreased [10, 11]. The *RET/PTC3* rearrangements were the most frequent ones during the “first wave” of PTC after the Chernobyl accident, while *RET/PTC1* seems to have predominated in cases with longer latency [13]. Old neglected cancers must have been particularly frequent among the early cases, when the pool of undiagnosed tumors was still untapped, equipment of histopathological laboratories not yet modernized, and post-Chernobyl radiophobia was at its apogee. The first wave PTC must have been on average “older” than those detected later [9]; accordingly, the former tumors were generally larger than the latter ones [14]. Furthermore, it was pointed out that *RET* rearrangements in the post-Chernobyl papillary PTC were associated with “increased aggressiveness in terms of pathological stage” (pT_4_ in particular) at the disease onset [15]. Considering uncertainties in regard to the disease onset, from this statement remains a quintessence: *RET* rearrangements in PTC were associated with increased aggressiveness in terms of pathological stage, the latter, in its turn, being naturally associated with disease duration. Moreover, it would be logical to assume that increased aggressiveness in terms of pathological stage would be associated with that in terms of histologic grade. It is therefore not surprising that the cancers developing after short latency were found to be “significantly less structurally differentiated” than later ones [14]. 

It is sometimes objected that screening cannot account for the age-related differences; there has been no clearly demonstrated increase in the incidence of cancers due to radiation from Chernobyl accident, except for the TC among patients exposed at a young age [16, 17]. In fact, there is an explanation: children at schools and kindergartens are easily available for screening; mass examinations of children and adolescents were performed by not always perfectly trained teams, under the conditions of high tumor expectancy. The following citations from a Russian-language professional publication provide some insight (verbatim from Russian): “Practically all nodular thyroid lesions, independently of their size, were regarded at that time in children as potentially malignant tumors, requiring an urgent surgical operation” or “Aggressiveness of surgeons contributed to the shortening of the minimal latency period” [18]. Although there was some overdiagnosis [5], many therapy-demanding cancers were successfully detected, so that the benefit from screening certainly prevailed. The role of screening (its ability to enhance registered TC incidence many times), of latent and dormant carcinomas [19] and tumors of uncertain malignant potential in overestimation of the post-Chernobyl TC incidence were discussed previously [5]. One of the mechanisms leading to overdiagnosis is as follows. If a thyroid nodule is found by screening, a fine-needle aspiration is usually performed. Aspiration cytology of the thyroid is accompanied by relatively high percentage of uncertain conclusions (so-called grey zone), when histological verification is indicated. Hemithyroidectomy or subtotal thyroidectomy was usually performed in such cases, and the surgical specimen was forwarded to a pathologist, who could be sometimes prone, after the in toto removal of the nodule, to confirm malignancy even in case of some uncertainty [6]. Our article describing this mechanism, possibly causing overdiagnosis and hypertherapy of TC even today, was rejected by the journal Arkhiv Patologii. Peculiarities of pediatric material added their share to uncertainty. The Head pediatric oncologist of Russian Federation professor Vladimir Poliakov pointed out shortage of cytologists, especially those having experience with pediatric material (personal communication, 2009). Foreign handbooks and atlases of cytology were seldom at working places. Note that indications for thyroidectomy on the basis of cytology alone should always be carefully considered, taking into account quality of cytological examination.

It was demonstrated that *RET* rearrangements can be induced by radiation in animal experiments and in vitro [20–22], but the doses were considerably higher than those in the setting of the Chernobyl accident. Correlations of *RET/PTC* rearrangements with radiation doses were found retrospectively among atomic bomb survivors in Japan [23, 24]. It is perfectly possible that *RET/PTC* rearrangements can be induced by ionizing radiation; however, in case of Chernobyl, as discussed above, the molecular-genetic features of the PTC were probably determined, in the first place, by factors other than radiation. Furthermore, high prevalence of *RET/PTC* rearrangements and reciprocally low prevalence of *BRAF* mutations were described as hallmarks of sporadic childhood PTC, unrelated to radiation [13]. Accordingly, it was assumed that the “waves” of PTC after the Chernobyl accident (first *RET/PTC3*, then *RET/PTC1*, and the third wave of *BRAF*-mutated PTC) represent a recapitulation of the age-related sequence observed in sporadic PTC [13]. The matter could be further clarified by correlation of *RET* rearrangements with disease duration, tumor size, histologic grade, and certain tumor phenotypes. An association of *RET-PTC3* with the less differentiated solid phenotype of PTC, which prevailed shortly after the accident, has already been demonstrated [11, 14]. It is in accordance with the hypothesis that the “first wave” tumors had on average higher histological grade because they were generally older and more advanced than those detected at a later date. In conclusion, the *RET* rearrangements, the *RET/PTC3* in particular, were probably related to the disease duration and a corresponding stage of tumor progression, and the “successive waves of tumors in those exposed to high levels of fallout as children, each with different molecular, morphological, and clinical findings” [10] after the Chernobyl accident were largely determined by changing approach to screening and diagnostics, their improvement with time, and exhaustion by screening of the pool of undiagnosed cancers. Finally, a short comment with regard to the nuclear plant accident in Fukushima after the greatest earthquake in the Japanese history is presented. The screening effect is obviously dependent on the basis level of medical surveillance: the higher the level, the smaller the screening effect. I am not well informed about conditions in Japan, but probably the basis level of medical checkups has been high and there will be no false registration of nonirradiated persons as victims of the Fukushima accident. There can be some increase of the registered TC after the accident due to the health examinations vigorously initiated in the Fukushima area, but it will be much less prominent than that after the Chernobyl accident. Absence of any significant increase of TC after the Fukushima accident in spite of the screening would certify the high level of health care in Japan especially among children and adolescents.
